# Genetic diversity and population structure of *Piper nigrum* (black pepper) accessions based on next-generation SNP markers

**DOI:** 10.1371/journal.pone.0305990

**Published:** 2024-06-26

**Authors:** Nilni A. Wimalarathna, Anushka M. Wickramasuriya, Dominik Metschina, Luiz A. Cauz-Santos, Dharshani Bandupriya, Kahandawa G. S. U. Ariyawansa, Bhathiya Gopallawa, Mark W. Chase, Rosabelle Samuel, Tara D. Silva

**Affiliations:** 1 Department of Plant Sciences, Faculty of Science, University of Colombo, Colombo, Sri Lanka; 2 Department of Botany and Biodiversity of Research, University of Vienna, Vienna, Austria; 3 Department of Botany, Faculty of Science, University of Peradeniya, Peradeniya, Sri Lanka; 4 Royal Botanic Gardens, Kew, United Kingdom; 5 Department of Environment and Agriculture, Curtin University, Perth, Western Australia, Australia; Universidade Federal do Para, BRAZIL

## Abstract

Despite the economic importance of *Piper nigrum* (black pepper), a highly valued crop worldwide, development and utilization of genomic resources have remained limited, with diversity assessments often relying on only a few samples or DNA markers. Here we employed restriction-site associated DNA sequencing to analyze 175 *P*. *nigrum* accessions from eight main black pepper growing regions in Sri Lanka. The sequencing effort resulted in 1,976 million raw reads, averaging 11.3 million reads per accession, revealing 150,356 high-quality single nucleotide polymorphisms (SNPs) distributed across 26 chromosomes. Population structure analysis revealed two subpopulations (*K* = 2): a dominant group consisting of 152 accessions sourced from both home gardens and large-scale cultivations, and a smaller group comprising 23 accessions exclusively from native collections in home gardens. This clustering was further supported by principal component analysis, with the first two principal components explaining 35.2 and 12.1% of the total variation. Genetic diversity analysis indicated substantial gene flow (*Nm* = 342.21) and a low fixation index (*F*_ST_ = 0.00073) between the two subpopulations, with no clear genetic differentiation among accessions from different agro-climatic regions. These findings demonstrate that most current black pepper genotypes grown in Sri Lanka share a common genetic background, emphasizing the necessity to broaden the genetic base to enhance resilience to biotic and abiotic stresses. This study represents the first attempt at analyzing black pepper genetic diversity using high-resolution SNP markers, laying the foundation for future genome-wide association studies for SNP-based gene discovery and breeding.

## Introduction

*Piper nigrum* L. (black pepper) is one of the oldest and most widely traded spices globally. The characteristic pungency and flavor of its dried mature fruits (berries) are largely due to the non-volatile alkaloid, piperine (1-piperoyl-piperidine). In addition to being a spice, black pepper possesses a range of pharmacological activities including antioxidant, antitumor [1, 2], antidepressant, anti-inflammatory, anti-diarrheal, analgesic [3], antibacterial, antifungal, anti-thyroid, anti-platelet formation and anti-hypersensitive activities [4–6].

*Piper nigrum* (2n = 52) is a perennial, climbing woody vine in Piperaceae which is the largest family in the order Piperales. It is believed to have originated in the humid tropical evergreen forests of the Western Ghats of Peninsular India [7]. From there, it spread to many tropical and subtropical regions so that, today, it is grown commercially in around 30 countries, mainly Vietnam (288,167 tons), Brazil (118,057 tons), Indonesia (81,219 tons), India (64,816 tons), Sri Lanka (42,465 tons) and Malaysia (31,636 tons) [8]. Despite relatively low production volumes, Sri Lanka produces some of the world’s finest black pepper types characterized by high levels of piperine. Thus, Sri Lankan black pepper, which is traded under the name “Ceylon black pepper”, fetches a premium price in the international spice market. Black pepper is an important cash crop, providing a significant source of income and employment for small-scale growers and rural communities in Sri Lanka and many other developing countries.

Sri Lanka possesses a rich genetic diversity of *P*. *nigrum*, both wild and cultivated varieties [9]. In the cultivated black pepper germplasm collection, various local selections, as well as two introductions, Panniyur-1 from India and Kuching from Malaysia, both introduced in the 1970s [10], are present. The crop is traditionally cultivated in the wet and intermediate agro-climatic regions with adequate rainfall, although it can be found in other geographical regions of the island as well. In anticipation of future climate scenarios, there is ongoing experimentation to expand black pepper cultivation beyond the conventional pepper-growing areas to drier regions of the island. Hence, the characterization of germplasm from these new localities will be important to uncover unique ecotypes, which is crucial for the development of superior, region-specific black pepper varieties that can thrive under diverse climate conditions.

Numerous studies have been conducted to assess the genetic diversity of black pepper germplasm in individual countries, with the aim of identifying the scope and depth of the available genetic resources for plant breeding. Previously, morphological markers have been used to characterize black pepper germplasm collections. However, morphometric analysis is time-consuming, and also not foolproof because of environmental influence on trait expression. Several previous studies have also evaluated the genetic diversity among black pepper accessions, mainly from India, using molecular markers including random amplified polymorphic DNA (RAPD) [11, 12], amplified fragment length polymorphism (AFLP) [13], and simple sequence repeats (SSRs) [12, 14, 15]. For instance, utilizing 24 RAPD markers, cultivar-specific bands were discerned among 09 advanced cultivars and 13 Indian landraces [11]. Another analysis employing 367 polymorphic RAPD markers detected greater genetic divergence among landraces, compared to advanced cultivars from South India [12]. Based on 158 polymorphic AFLP markers [13], 30 Indian black pepper cultivars were grouped into three major clusters and four unique cultivars. These investigations revealed complex and extensive genetic diversity present in black pepper germplasm. However, a notable limitation in many of these investigations is their reliance on a limited number of samples and markers, which may not provide a comprehensive understanding of black pepper genetic diversity.

In a new development, Hu et al. [16] published the first chromosome-scale reference genome assembly of black pepper, facilitating the discovery and characterization of genome-wide SSRs. Using this reference genome, 50 genome-wide SSR markers were developed that discriminated 30 Indian black pepper landrace accessions in four distinct clusters [17]. More recently, Negi et al. [18] characterized chromosomal location-specific polymorphic SSRs from 29 Indian black pepper accessions, with potential application in diversity analysis. Although SSR markers have been the most widely used for assessing the genetic diversity of crops, the characterization of large germplasm collections by this method could be time-consuming and expensive. Additionally, the results may not completely reflect the relatedness among individuals across all genomic regions, limiting their application in population genomics studies.

Next-generation sequencing (NGS) technologies have provided an unprecedented opportunity to study population genetics with increased efficiency at a much higher resolution [19, 20] in diverse organisms, including agriculturally important plants, allowing for the discovery of genome-wide molecular marker data, particularly markers based on single nucleotide polymorphisms (SNPs), with increased efficiency and at a much higher resolution. SNPs represent the most abundant form of genetic variation in a species and generally exhibit low mutation rates. In addition, SNPs are amenable to high throughput detection platforms, allowing rapid analysis of large data sets. In recent years, SNP markers have been used to evaluate the genetic diversity of many important crops including rice [21], oil palm [22] and barley [23].

Although whole-genome sequencing of a large, diverse set of individuals would capture rare and low-frequency SNPs, detection of SNPs through complete genome sequencing of several hundred individuals would still be expensive [24]. Therefore, several high-throughput, and cost-effective, reduced-representation, sequencing-based genotyping methods have been developed in the last decade. These methods allow the discovery of thousands of polymorphic genetic markers randomly distributed across the genome. One of the best techniques for reduced-representation sequencing-based genotyping is restriction site-associated DNA sequencing (RADseq) [25–28], which is particularly useful for non-model organisms with either no or limited genomic resources. RADseq has been extensively used in generating genome-wide molecular data for species delimitation [29], phylogeography [30–32], phylogenetic reconstruction [31], population genomics [33, 34], and ecological studies [35].

Despite the development of improved commercial cultivars, we hypothesize that there remains untapped genetic diversity within the Sri Lankan black pepper germplasm. Thus, our study aimed to examine samples from both commercial plantations and home gardens across different agro-climatic regions. Our specific objective was to develop a set of high-quality RADseq-derived SNP markers and utilize them to reveal population structure and diversity across a range of germplasm accessions. To date, SNP-detected genetic diversity and population structure analyses have not been performed for black pepper. Furthermore, molecular markers have not been used to explore the diversity in the Sri Lankan black pepper germplasm previously. Therefore, our study addressed this dual research gap, presenting a pioneering investigation into the utilization of SNP markers for the characterization of Sri Lankan black pepper germplasm.

## Materials and methods

### Sample collection and genomic DNA extraction

**Fig 1:**
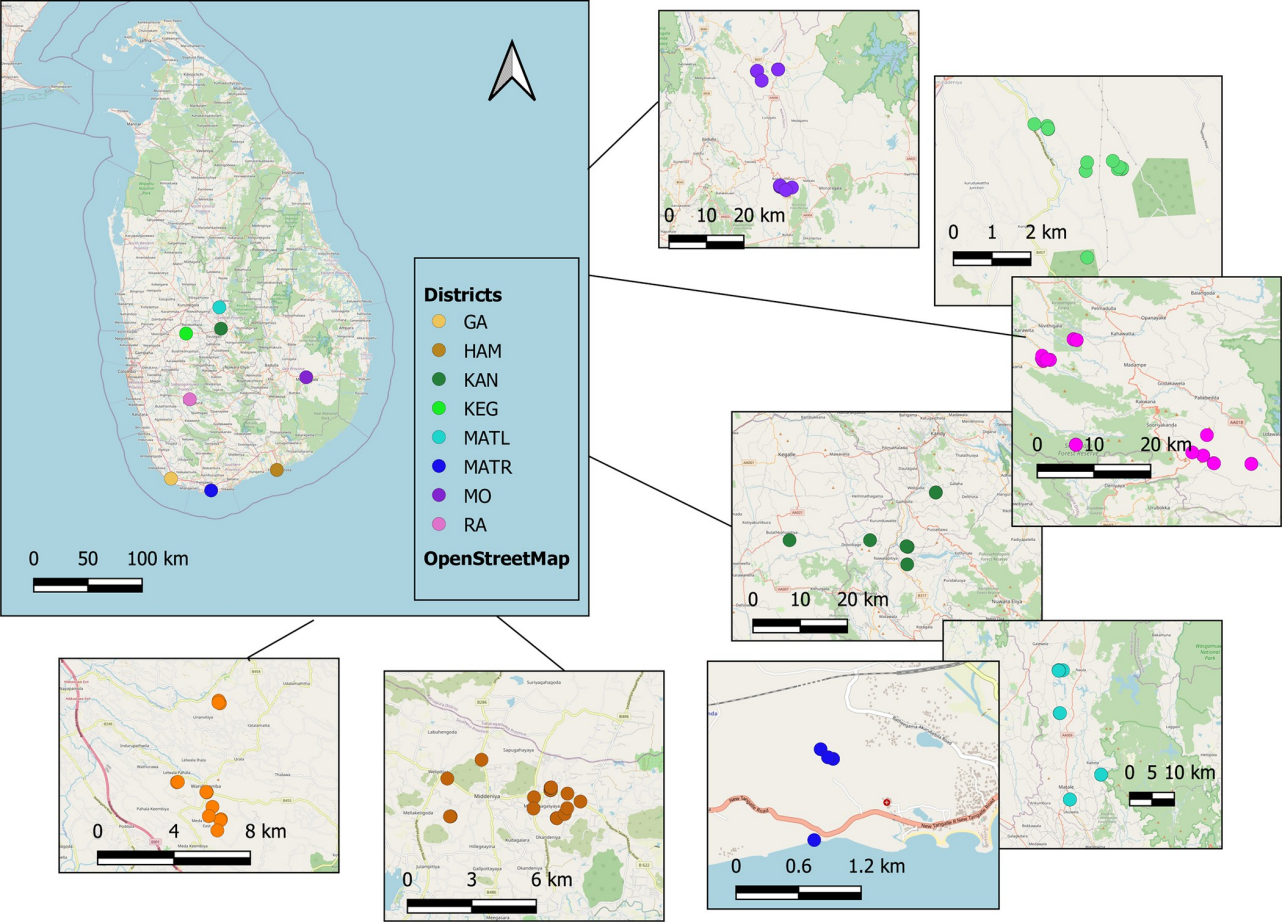
Sites of collection of *Piper nigrum* accessions. The collection sites of *P*. *nigrum* accessions in each region are given in the insets: GA: Galle; HAM: Hambantota; KAN: Kandy; KEG: Kegalle; MATL: Matale; MATR: Matara; MO: Moneragala; RA: Ratnapura. The map was generated using QGIS v.3.28.3, and contains information from OpenStreetMap and OpenStreetMap Foundation, available under the Open Database License.

Leaf samples were collected from 175 accessions of black pepper, from large plantations and home gardens, in eight Sri Lankan agro-climatic regions (districts): Galle, Matara, Hambantota, Ratnapura, Monaragala, Kegalle, Kandy, and Matale (Fig 1, S1 Table). The voucher specimens were identified by Nilni Wimalarathna and deposited at the National Herbarium of the Department of National Botanic Gardens, Sri Lanka. Permission to collect plant samples for this research was provided by the Department of Wildlife Conservation, Sri Lanka (WL/3/2/56/21), the Department of Forest Conservation, Sri Lanka (FD/EX/50/Nor/2022/02/20), and the Department of Export Agriculture, Sri Lanka (DGO/04/01). Fresh, immature leaves from each accession were harvested and dried in silica gel. From each accession, approximately, 20 mg dried leaves were ground to a fine powder using a TissuelLyser II (Qiagen, Germany). Total genomic DNA was extracted using the DNeasy Plant Mini Kit (Qiagen, Germany) according to the manufacturer’s instructions.

### RADseq library preparation and sequencing

Libraries for RADseq were constructed by adapting a protocol from previous studies, with modifications [26, 36–39]. In brief, from each individual, 200 ng of intact, high-quality genomic DNA was separately digested with PstI (New England Biolabs (NEB), USA) for 2 h at 37°C in a 50 μl reaction volume containing 1 μl of PstI and 5 μl of 10X 3.1 buffer (NEB, USA). PstI was heat-inactivated by incubation at 80°C for 20 min. For the ligation reaction, 4 μl of barcoded P1 adapters (300 nM), 3.5 μl of nuclease free water, 0.5 μl T4 DNA ligase (NEB, USA), 1 μl of NEBuffer (NEB, USA) and 1 μl of rATP (100 mM) (Promega, USA) were added to the digested products and the samples were incubated overnight at 16°C. The ligation reactions were inactivated by incubating the samples at 65°C for 10 min. The adapter-ligated products were then pooled and randomly sheared by sonication using a Bioruptor Pico (Diagenode, Belgium) with two cycles of 45s ON/60s OFF at 4°C, to yield DNA fragments of approximately 400 bp. The sheared DNA was then purified with MinElute^R^ Reaction Cleanup Kit (Qiagen, Germany) followed by left (0.15x) and right (0.55x) side size selection with SPRIselect Reagent Kit (Beckman Coulter, USA). The ends of the fragments were repaired using Quick Blunting Kit (NEB, USA) and purified with MinElute^R^ Reaction Cleanup Kit (Qiagen, Germany). Subsequently, an adenosine tail was added to the end-repaired fragments (15 μl) using 2 μl of Klenow fragment 3 -> 5 exo^-^ (5000 U/ml) (NEB, USA), 2 μl NEBuffer (NEB, USA) and 1 μl of dATP (100 mM) for 30 minutes at 37°C. The end-repaired, adenylated DNA fragments were then ligated to P2 adapters, purified, and size selected in sequential steps.

The DNA fragments containing both P1 and P2 adapters were enriched via polymerase chain reaction (PCR) and purified with MinElute^R^ PCR Purification Kit (Qiagen, Germany). RAD libraries were sequenced on the Illumina NovaSeq SP PE150 platform to generate 150 bp pair-end (PE) reads at the Next Generation Sequencing Facility at Vienna BioCenter Core Facilities (VBCF NGS Unit) (www.vbcf.ac.at). The sequence reads generated for this study are available in the National Center for Biotechnology Information (NCBI) Sequence Read Archive under BioProject accession ID PRJNA1035754 (https://dataview.ncbi.nlm.nih.gov/object/PRJNA1035754?reviewer=sd5vgms9c964a2jfvfdm5rimg9).

### Data processing and variant filtering

The data were demultiplexed into sub-libraries based on index barcodes using BamIndexDecoder v.1.03 of the Picard Illumina2Bam package (http://gq1.github.io/illumina2bam/). The bam files in each sub-library were converted to paired-end fastq files using SamToFastq tool included in Picard tools v.2.18.26 (https://github.com/broadinstitute/picard/). Subsequently, the reads were further demultiplexed to individuals based on their inline barcodes with *process_radtags* in Stacks v.1.47 [40]. Simultaneously, the reads with low-quality scores and uncalled bases were removed while retaining barcodes with a maximum of one mismatch.

The processed reads were aligned with the reference genome of *P*. *nigrum* (GenBank Bio-project number: PRJNA529758) [16] using Burrows-Wheeler aligner with its maximal exact match (BWA MEM) algorithm (v.0.7.17) [41]. During this step, the–M option was used to flag shorter split hits as secondary, and mapping statistics and coverage were obtained. The sequence alignment map (SAM) files were indexed, and sorted by reference coordinate, and read groups were added with the Picard Toolkit (http://broadinstitute.github.io/picard/). Indel realignment was performed using the Genome Analysis Toolkit (GATK) v.3.8.1 [42] to improve the mapping quality around indels, thinning the data to a maximum of 100,000 reads per interval.

GATK v.3.8.1 was applied to identify variants following the recommendations of GATK best practices [43]. In brief, the genomic variant call format (GVCF) mode of HaplotypeCaller was used to generate an intermediate file per sample with a.g.vcf extension, and subsequently, all GVCF files were genotyped together by the GenotypeGVCFs module. The variants present in at least 50% of the individuals were retained using VCFtools v.0.1.16 [44]. The generated variant call format (VCF) file was further filtered with the VariantFiltration module in GATK using the following criteria: (1) depth of coverage (DP) < 500; (2) variant confidence (QUAL) < 30.00; (3) variant confidence divided by the unfiltered depth (QD) < 2; (4) Phred-scaled P-value for the Fisher’s exact test to detect strand bias (FS) > 60; (5) a root mean square of mapping quality across all samples (MQ) < 40; (6) u-based z-approximation from the rank sum test for mapping qualities (ReadPosRankSum) < -8.0; and (7) u-based z-approximation from the rank sum test for the distance from the end of the reads with the alternate allele (MQRankSum) < -12.5. Additional filtering of variants was performed using VCFtools v.0.1.16. to retain SNPs with a minor allele frequency (MAF) ≥ 0.011 (i.e., present in at least four haplotypes), an average depth above 25, and a maximum of 20% missing data. Moreover, the program populations from Stacks were used to retain variable positions with a maximum observed heterozygosity of 0.65, to avoid using any pooled paralogs [45]. The distribution of filtered SNPs along the chromosomes was plotted using a density map tool (https://www.bioinformatics.com.cn) [46].

### Analysis of genetic diversity and population structure

To infer the genetic relatedness between accessions of *P*. *nigrum*, a coancestry heatmap was constructed using Analysis of Next Generation Sequence Data (ANGSD) v.0.930 [47]. For this purpose, indel-realigned mapping files were used as input files to calculate genotype likelihoods in the genotype-free variant calling method implemented in ANGSD. Only the variable sites that were detected in at least 50% of individuals and reads with a minimum of 20 base and mapping quality were used. The covariance matrix was calculated from the genotype likelihoods using principal component analysis of NGS data (PCAngsd) [48] and visualized as a coancestry heatmap using the heatmaps.2 function in Gplots v.3.1.3 (http://CRAN.R-project.org/package=gplots) [49] in Rstudio v.4.3.1 [50, 51]. In addition, principal component analysis (PCA) was performed based on the covariance matrix and visualized using R package Gplots.

Furthermore, from the estimated genotyped likelihoods in ANGSD, we selected unlinked sites with 10,000 bp between variants, and population structure was inferred using NGSadmix [52]. Ten independent runs were performed by setting the number of populations (*K*) to range from one to ten. The Evanno’s Δ*K* (difference in likelihood between adjacent K values) method [53] was used to determine the best *K*-value for estimating the optimal number of subpopulations within the accessions using Clustering Markov Packager Across K (CLUMPAK, http://clumpak.tau.ac.il/bestK.html) and visualized using Rstudio v.4.3.1.

Moreover, variants identified in GATK v.3.8.1 with additional filtering in VCFtools v.0.1.16, were used to estimate the genetic diversity of the subpopulations. Gene diversity, inbreeding coefficient (*F*_IS_), nucleotide diversity (Π), observed heterozygosity (*H*o), expected heterozygosity (*H*e), percentage of polymorphic loci (*PPL*), and the number of private alleles (*N*_P_) per group were estimated using *fstats* module in the populations program of the Stacks v.1.47 package [40]. Polymorphism information content (PIC) was calculated as described by Botstein et al. [54]. Analysis of molecular variance (AMOVA) [55] was performed for structure-based subpopulations using Arlequin ver. 3.5.2.2 [56], and the fixation index (*F*_ST_) was calculated to estimate the genetic differentiation between subpopulations. The statistical significance of AMOVA was evaluated using 1,000 permutations. The gene flow (*Nm*) between subpopulations was indirectly estimated by a traditional method based on genetic differentiation *Nm* = (1 –*F*_ST_)/4*F*_ST_ [57].

### Phylogenetic tree analysis

To perform the phylogenetic tree analysis, the final filtered VCF file was converted to PHYLIP format using PGDSpider v.2.1.1.3 [58]. The script ascbias.py (https://github.com/btmartin721/raxml_ascbias) was used to remove invariant sites from the alignment. This retained a total of 150,356 variant sites. With *P*. *fallax* as the outgroup, maximum likelihood (ML) analysis was performed in Randomized Axelerated Maximum Likelihood (RAxML) v.8.2.12 [59] with the general time reversible substitution model and CAT approximation of rate heterogeneity (GTRCAT) model, along with a recommended ascertainment bias correction of the likelihood [60] and 1,000 rapid bootstrap replicates. The best-scoring ML tree resulting from this analysis was visualized in FigTree v.1.4.4 (http://tree.bio.ed.ac.uk/software/figtree/).

## Results

### RAD sequencing and SNP discovery

Sequencing of DNA fragments from 175 *P*. *nigrum* accessions on the Illumina NovaSeq SP PE150 platform generated approximately 289 Gb sequence data containing 1,976,094,274 PE sequence reads. The number of reads generated varied from 1,619,259 to 20,815,475 with an average of 11,291,967 reads per accession (S2 Table). The number of reads mapped to the *P*. *nigrum* reference genome varied from 1,451,634 to 19,427,551 with an average of 10,676,528 reads per accession. The mapping coverage also varied between the accessions and ranged from 5.10 to 35.50. The average mapping coverage of accessions was 20.53 (Table 1).

**Table 1:** Mapping statistics of 175 *Piper nigrum* accessions.

	Mapped reads (%)	Mapping coverage	Paired-end reads	Both pairs mapped	Proper pairs	Singletons
Minimum	82	5.10	1,619,259	1,153,947	1,357,190	6,697
Maximum	99	35.50	20,815,475	19,376,625	18,594,765	171,267
Average	95	20.53	11,291,968	10,655,893	10,132,312	39,550

The number of singletons varied from 6,697 to 171,267 with an average of 39,550. Initially, after the variant calling and filtering in GATK, we identified a total of 4,340,399 variants. After additional filtering with VCFtools, which allows a maximum *H*o of 0.65, and a maximum 20% missing data per locus, only 150,356 variable sites were retained for further analysis.

**Fig 2:**
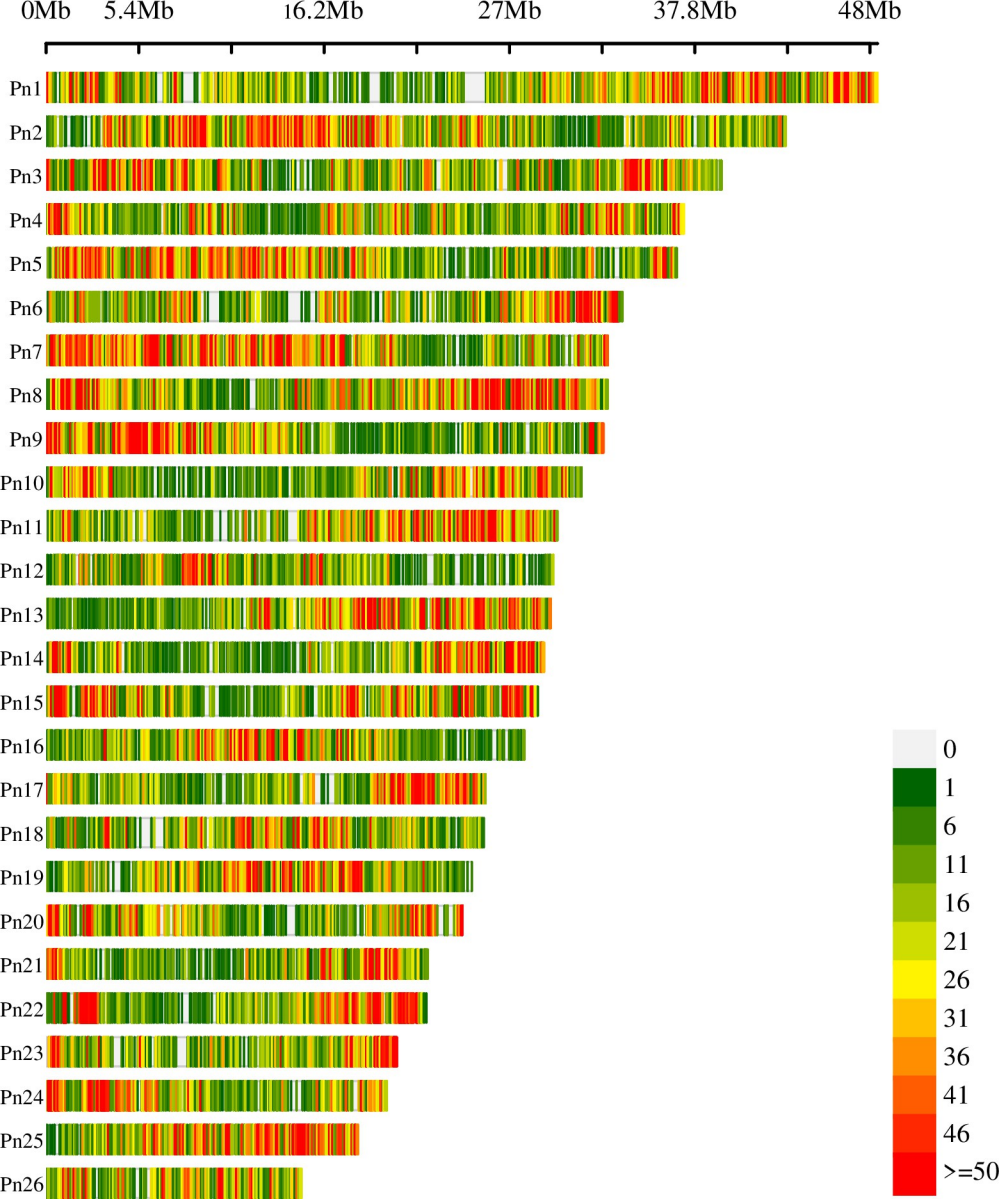
SNP density plot. The distribution of 150,329 SNP markers within 0.1 Mb window size across 26 chromosomes. Colored bars are SNP counts in 0.1 Mb interval.

A total of 150,329 filtered SNPs occurred on all 26 chromosomes of the *P*. *nigrum* genome (Fig 2). The remaining 27 SNPs were distributed across scaffolds 27 to 45. SNPs were grouped into 0.1 Mb window sizes and mapped onto 26 chromosomes and exhibited a clustered pattern. The greatest number of SNPs was observed on chromosome 2 (9,260) and the lowest on chromosome 26 (2,948), an average of 5,782 SNPs per chromosome. An average of 20 SNPs was detected per 0.1 Mb. The highest SNP density (> 28 SNPs/0.1 Mb) was observed on chromosome 25 and the lowest on chromosome 12 (< 13 SNPs/0.1 Mb).

### Population structure analysis

**Fig 3:**
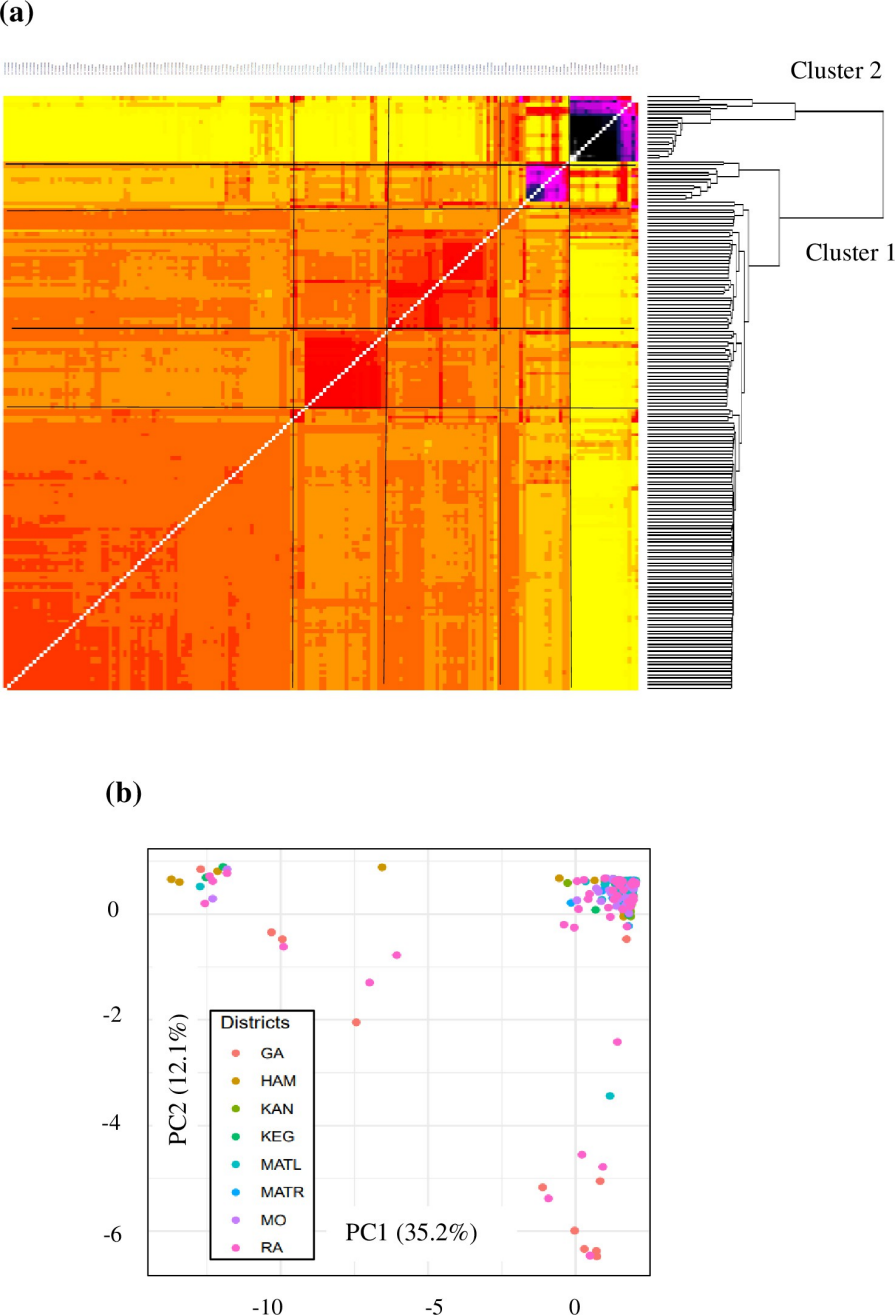
Clustering patterns of 175 *Piper nigrum* accessions based on ANGSD. (a) Co-ancestry heatmap with covariance matrix results from PCAngsd. Identities of accessions used in the study are provided in the supporting information (S1 Table). Clustering patterns of *P*. *nigrum* accessions are shown to the right. Diagonal estimation is excluded. (b) PCA based on 4,089,675 polymorphic sites from PCAngsd. Districts are represented in different colors. GA: Galle; HAM: Hambantota; KAN: Kandy, KEG: Kegalle; MATL: Matale; MATR: Matara; MO: Moneragala; RA: Ratnapura.

ANGSD retained 4,089,675 variable sites from a total of 417,152,846 reviewed sites. Based on the covariance matrix results obtained from PCAngsd, two main genetic groups were identified in the heatmap with the larger cluster exhibiting four sub-clusters (Fig 3A). Accessions collected from the eight regions were represented in the two main clusters and the four sub-clusters, with no clear genetic separation based on geographic region or district. The coordinates of the PCA explained 35.2% and 12.1% of the total variation (Fig 3B).

**Fig 4:**
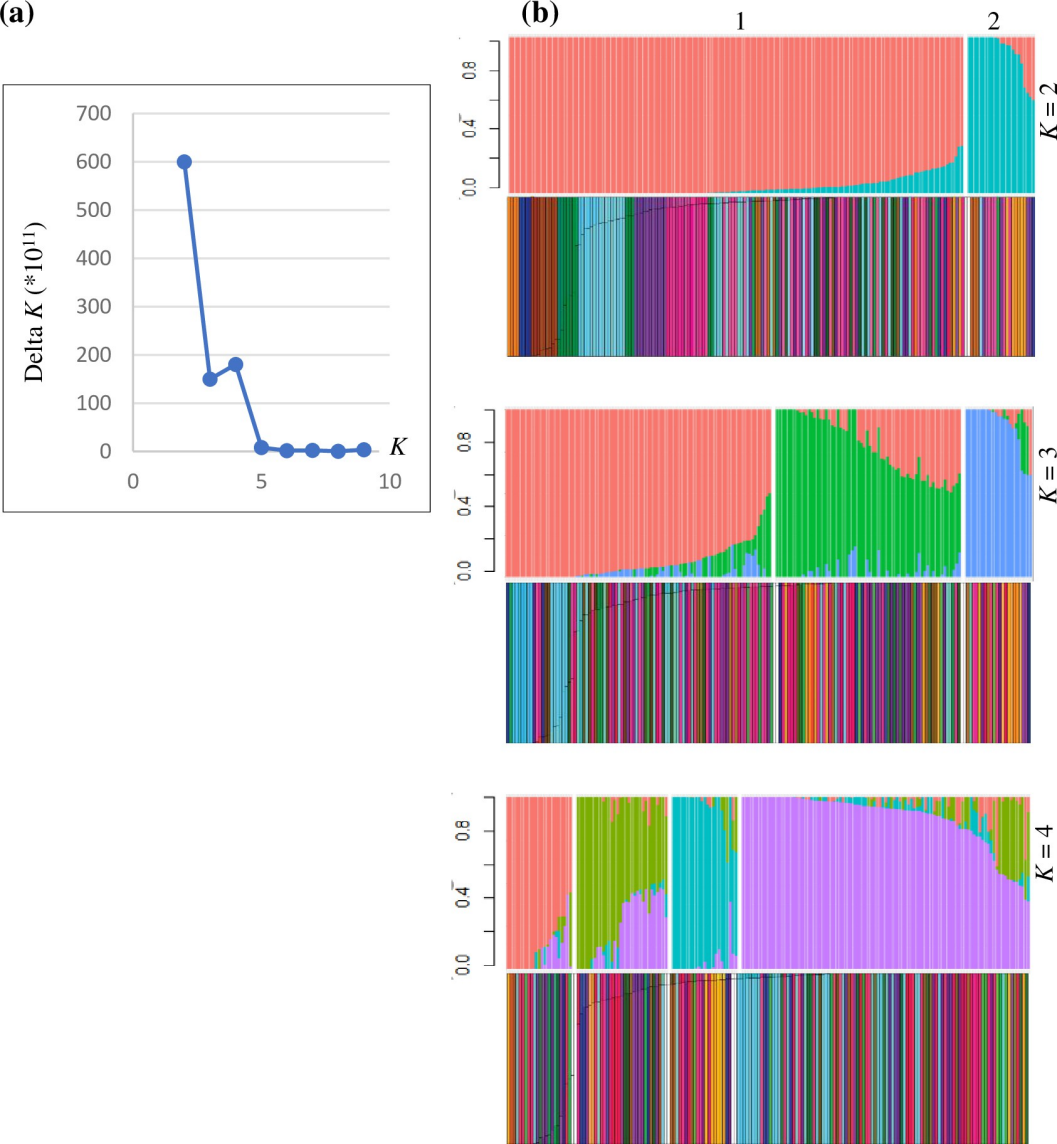
Genetic structure of 175 *Piper nigrum* accessions from eight agro-climatic regions. (a) Delta *K* values for different numbers of populations assumed in NGSadmix analysis (b) Genetic structure and admixture based on 37,341 unlinked polymorphic sites from ANGSD. Admixture diagrams show *K* = 2, 3, and 4. In the admixture diagrams, each vertical line represents one individual, and each color represents one inferred ancestral population. The length of each color in a vertical bar represents the proportion of that individual’s ancestry to the ancestral population corresponding to that color. Subpopulations at *K* = 2 are shown on top of the admixture diagram. Color coordinated district diagrams corresponding to the admixture plots are shown below each admixture diagram. Each vertical bar represents one individual accession and each color represents one district. Colors are the same as in the PCA.

For population structure inference in NGSadmix, 37,341 unlinked sites with a minimum distance of 10,000 bp were retained. Structure analyses were performed with *K* values ranging from 1 to 10, with 10 runs performed for each *K* value (Fig 4A). The highest Δ*K* was observed at *K* = 2, indicating the optimal inferred populations within the set of 175 *P*. *nigrum* accessions. The larger subpopulation included 152 *P*. *nigrum* accessions (92%) from all eight regions (Fig 4B). Notably, as *K* values increased, additional structure was revealed within the larger cluster, while the smaller subpopulation remained uniform. At *K* = 2, 20 *P*. *nigrum* accessions (11%) exhibited admixture.

**Fig 5:**
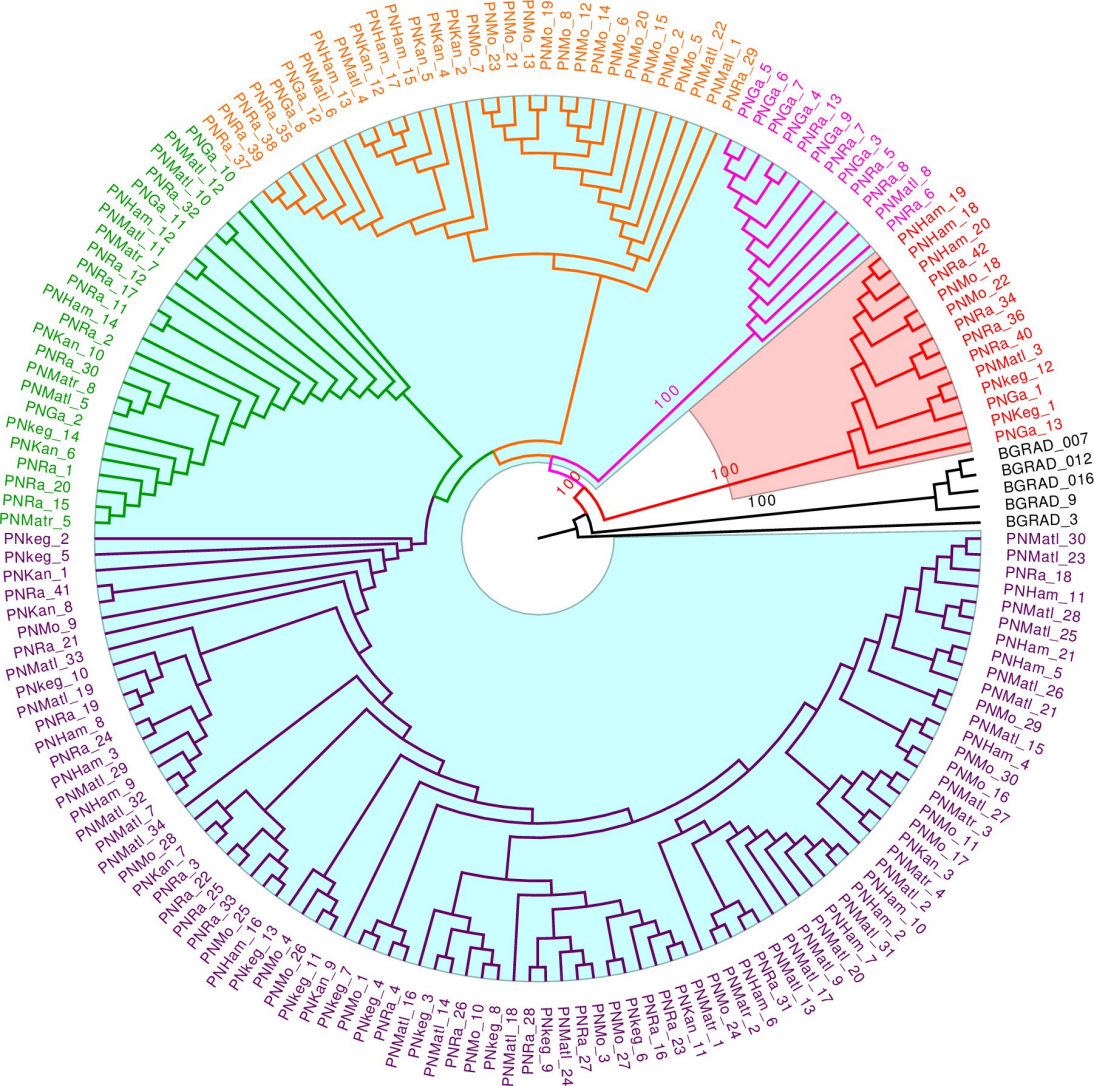
SNP-based ML phylogenetic tree constructed for 175 *Piper nigrum* accessions. Bootstrap percentages are shown on the branches. *Piper fallax* (BGRAD_3, BGRAD_007, BGRAD_9, BGRAD_012, and BGRAD_016) was chosen as the outgroup to root the tree. Two main clusters (cluster 1 and 2) in the ML tree are highlighted in blue and red, respectively. Four sub-clusters within cluster 1 are shown in different colors: purple, green, orange and pink.

The larger cluster included accessions collected from both home gardens and large-scale cultivation. In contrast, cluster 2 (red, Fig 5) consisted of only those collected from home gardens. In the larger cluster, four sub-clusters were observed, each consisting of accessions from five or six of the eight districts, except one (pink, Fig 5) that was clearly separated from the others and included individuals from only two districts (Galle and Ratnapura). The only non-native accession, Panniyur-1 (PNRa_17), grouped in one sub-cluster (green, Fig 5) along with the three hybrids; Dingi_Rala (PNMatl_10), Bootawe_Rala (PNMatl_11), and Kohukumbure_Rate_Rala (PNMatl_12), produced from crosses between Panniyur-1 and three local accessions (GK49, DM7 and MW21). The results of the larger groupings in phylogenetic analysis were consistent with those in the heatmap and PCA.

### Genetic diversity analysis

The analysis of genetic diversity parameters for the entire population showed that average gene diversity, *H*o, and MAF were 0.130, 0.103, and 0.078, respectively. The *H*e ranged from 0.023 to 0.500, with an average of 0.129. The average Π was 0.130, ranging from 0.023 to 0.503. Moreover, analysis of genetic diversity parameters between the two subpopulations showed that the average values of gene diversity, *H*o and *H*e were 0.134, 0.103, and 0.132, respectively. The average *N*p was 10,124. Except for the higher number of *N*p, genetic diversity values in subpopulation 1 (*H*o = 0.102, *H*e = 0.128) were similar to subpopulation 2 (*H*o = 0.104, *H*e = 0.136). The average *PPL* was 91.79%, ranging from 83.60% in subpopulation 2 to 99.97% in subpopulation 1. The Π ranged from 0.129 in subpopulation 1 to 0.139 in subpopulation 2. The PIC values of subpopulation 1 and 2 were 0.128 and 0.136, respectively. The intra-population *F*_IS_ values ranged from 0.203 (subpopulation 2) to 0.237 (subpopulation 1) with an average of 0.220 (Table 2), indicating a deficiency of heterozygotes compared with the expected Hardy-Weinberg proportions.

**Table 2:** Genetic diversity measurements of structure-based two subpopulations of *Pipernigrum* accessions based on 150,356 SNP markers.

Subpopulation	N	*N*p	*H*o	*H*e	*PPL* (%)	Π	PIC	*F*_IS_
1	152	20,208	0.102	0.128	99.97	0.129	0.128	0.237
2	23	40	0.104	0.136	83.60	0.139	0.136	0.203
Average		10,124	0.103	0.132	91.79	0.134	0.132	0.220

N, number of accessions; *N*p, private alleles; *H*o, observed heterozygosity; *H*e, expected heterozygosity; *PPL*, percentage of polymorphic loci; Π, nucleotide diversity; PIC, polymorphism information content; *F*_IS_, inbreeding coefficient.

Based on AMOVA (S3 Table), pair-wise genetic differentiation between the two subpopulations was significantly low (*F*ST = 0.00073, p < 0.0001) with an estimated gene flow of 342.21 migrants per generation. A large proportion of the total variation was attributed to genetic differences within subpopulations (99.93%) rather than between the two subpopulations (0.07%).

## Discussion

The center of origin of *P*. *nigrum* has been presumed to be the Western Ghats in southern India, although no accessions or species from Sri Lanka were included in previous studies. Kerala State in south-western India has been identified as the region with the highest genetic diversity of wild accessions of black pepper [61]. Sri Lanka was connected to southern India before separating as an island. The shared geological history and close affinities of flora in the two regions have led to their joint recognition as a unified entity within the current classification of global biodiversity hotspots [62]. Nevertheless, the high level of endemism found in the island suggests that geographic isolation and unique evolutionary pressures have created conditions for the evolution of distinct species and ecotypes in the island [62, 63]. Therefore, studying the genetic diversity of black pepper in Sri Lanka is important for conservation and crop improvement efforts, because it potentially can lead to identification of important traits valuable for breeding programs.

Several studies have been conducted on the characterization of region- or country-specific black pepper germplasm, particularly from India [64], China [15], Indonesia [65, 66] and Malaysia [67]. Although some studies used classical morphological markers, many were based on DNA markers, relying primarily on RAPD [11, 12], AFLP [13], and SSRs [12, 14, 15], but there have been no reports thus far on the utilization of SNP markers for genetic diversity studies in black pepper. Furthermore, the availability of genome-wide molecular data for black pepper has remained limited until recently [68, 69]. Although SNP-based analyses of genetic variation and population structure have been reported in several crop species such as rice [70], maize [71], sorghum [72], tomato [73], and tea [74], this is the first report on the utilization of SNP markers for genetic diversity studies in black pepper.

Here the high-quality SNPs were found across all 26 chromosomes. Compared to previous studies that relied on PCR-based markers, our research uncovered a greater number of SNPs, enabling more robust and comprehensive analyses of population structure and genetic diversity. In our study, the 175 accessions were divided into two main clusters (*K* = 2), with further fine-scale structuring observed within the larger cluster. However, these clusters did not correspond to geographical locations. The larger cluster (subpopulation 1) comprised cultivars from all eight localities, indicating genetic similarity among accessions from different agro-climatic regions. The lack of geographic structuring is likely due to human-mediated activities. The common practice of sharing popular cultivars among neighbors, friends, and family likely facilitated the distribution of favored germplasm across wide geographic and climatic ranges. Subpopulation 1 comprised several improved *P*. *nigrum* cultivars, including Panniyur-1 (from South India) and three hybrids (Panniyur-1 x local selections) bred for superior yields. The genetic similarity between Panniyur-1 and local cultivars highlights the relatedness of the germplasm between the two regions.

Currently, black pepper is cultivated in Sri Lanka on both plantation scale and in home gardens, which we sampled here. Large-scale plantations primarily consist of newly developed cultivars from selective breeding, whereas small-scale growers often maintain traditional cultivars and landraces passed down through generations. The presence of some accessions from home gardens in subpopulation 1 indicates that small-scale growers, recognize the economic advantages of these new cultivars and, have adopted them for their home plots. Conversely, the smaller cluster (subpopulation 2) primarily consisted of *P*. *nigrum* accessions sourced from home gardens, likely including traditional cultivars and landraces. These findings are consistent with the absence of geographic clustering observed in the results of the ML-based phylogenetic tree analysis and PCA. The first two PCA axes together explained 47.3% of the total genetic variation among black pepper genotypes, highlighting the potential of SNP markers for genetic diversity studies in black pepper. The observed heterozygosity (*H*o) in subpopulation 1 (0.102) and subpopulation 2 (0.104) was very low, a result compatible with their natural high frequency of self-pollination, in both traditional and improved races, a result of selection during crop domestication. Only the wild progenitors are known to be cross-pollinated [75]. The expected heterozygosity (*H*e) based on Hardy-Weinberg expectations (HWE) would be higher. Consistent with this, the average *H*e (0.132) for the overall population was higher than the average *H*o (0.103). The discrepancy between *H*o and *H*e implies a high level of inbreeding among individuals in the population, leading to reduced genetic diversity. Heterozygote deficiency is also indicated by the positive inbreeding coefficient (average *F*_IS_ = 0.220). Furthermore, the low fixation index (*F*_ST_ = 0.00073) and high gene flow (*Nm* = 342.21) suggest a low level of genetic differentiation and a significant level of genetic exchange [76] between the two subpopulations. This was also evident in the AMOVA results, which identified variance between subpopulations to be very low (0.07%) compared to very high variance within subpopulations (> 99%).

Private alleles (*N*p), which represent genetic variants unique to a particular population [77], were much more numerous in subpopulation 1 (20,208) compared to subpopulation 2 (40). However, this discrepancy is likely due to the larger number of genotypes clustered in subpopulation 1 (152) compared to subpopulation 2 (23), rather than an indication of greater allelic richness in subpopulation 1. The ability of a molecular marker to detect polymorphism, often quantified by its PIC, is a valuable parameter for assessing the informativeness of markers in genetic studies, particularly in genetic diversity and linkage analysis [78–81]. Multi-allelic co-dominant markers, such as SSRs, can have PIC values ranging from 0 to 1, with highly informative markers typically having values greater than 0.5. In contrast, SNP markers have a maximum PIC value of 0.5 due to their bi-allelic nature [79, 82–85]. Our results showed an average PIC value of 0.132, indicating that the SNP markers identified in black pepper are moderately informative. Even though this contrasts with previous studies on black pepper diversity, where SSR markers yielded higher PIC values of 0.85 [64] and 0.93 [15], SNP markers allow for the identification of allelic diversity across numerous loci, thus enhancing their utility in comprehensive genetic assessments [86].

Genetic diversity in the sampled accessions of *P*. *nigrum* was relatively low compared to the previous genetic analyses carried out using RAPD [11], AFLP [13], and SSR [15, 18] markers. The accessions used in this study were those in cultivation at present, which have been subjected to different selective pressures, particularly for improved yield and quality characteristics. Although subpopulation 2 may still represent a useful genetic resource for future black pepper breeding, it is essential to explore wild germplasm where the gene pool has not been influenced by human-mediated gene flow and selection, and unique ecotypes have had the opportunity to develop in isolation. This could uncover genetic diversity crucial for the development of resilient and high-performing cultivars.

## Conclusions

In this study, we employed RADseq technique to generate 150,356 high-quality SNPs for exploring genetic diversity and population structure in a collection of 175 Sri Lankan black pepper accessions. Our results demonstrate that Sri Lankan black pepper cultivars exhibit a narrow genetic base, indicating limited sampling of the total gene pool during cultivar development. This could seriously compromise the evolutionary potential of black pepper unless breeding and conservation strategies are implemented to enhance its genetic diversity. Additionally, this study identified two distinct subpopulations in sampled black pepper accessions, with subpopulation 2 solely comprising home garden collections, likely representing unique genetic resources. Notably, subpopulation 1, which had the largest number of accessions, exhibited the highest values for *N*p and *PPL*. The results of this study can serve as a baseline for future genetic studies, particularly in genome-wide association studies and marker-assisted selection targeting economically useful traits in black pepper.

## Supporting information

S1 Table Name, district, and the location of 175 *Piper nigrum* accessions. (PDF)

S2 Table The summary statistics for quality filtered reads of 175 *Piper nigrum* accessions. (PDF)

S3 Table Analysis of molecular variance (AMOVA) among structure-based two populations of *Piper nigrum* accessions. (PDF)
