# Supplementary material for: Genetic diversity and population structure of Piper nigrum (black pepper) accessions based on next-generation SNP markers
Source: PLoS One. 2024 Jun 26;19(6):e0305990. doi: 10.1371/journal.pone.0305990 (PMC11207170; doi:10.1371/journal.pone.0305990)
Supplement: S1 Table — (PDF) [file pone.0305990.s001.pdf]

S1 Table Name, District, and the location of 175 *Piper nigrum* accessions

Accession	District	Location	Accession	District	Location
PNGa_1	Galle	Home garden	PNMatl_32	Matale	Large scale cultivations
PNGa_2	Galle	Home garden	PNMatl_33	Matale	Large scale cultivations
PNGa_3	Galle	Home garden	PNMatl_34	Matale	Large scale cultivations
PNGa_4	Galle	Home garden	PNKan_1	Kandy	Home garden
PNGa_5	Galle	Home garden	PNKan_2	Kandy	Home garden
PNGa_6	Galle	Home garden	PNKan_3	Kandy	Home garden
PNGa_7	Galle	Home garden	PNKan_4	Kandy	Home garden
PNGa_8	Galle	Home garden	PNKan_5	Kandy	Home garden
PNGa_9	Galle	Home garden	PNKan_6	Kandy	Home garden
PNGa_10	Galle	Home garden	PNKan_7	Kandy	Home garden
PNGa_11	Galle	Home garden	PNKan_8	Kandy	Home garden
PNGa_12	Galle	Home garden	PNKan_9	Kandy	Home garden
PNGa_13	Galle	Home garden	PNKan_10	Kandy	Home garden
PNGa_14	Galle	Home garden	PNKan_11	Kandy	Home garden
PNMatr_1	Matara	Home garden	PNKan_12	Kandy	Home garden
PNMatr_2	Matara	Home garden	PNMo_1	Moneragala	Home garden
PNMatr_3	Matara	Home garden	PNMo_2	Moneragala	Home garden
PNMatr_4	Matara	Home garden	PNMo_3	Moneragala	Home garden
PNMatr_5	Matara	Home garden	PNMo_4	Moneragala	Home garden
PNMatr_6	Matara	Home garden	PNMo_5	Moneragala	Home garden
PNMatr_7	Matara	Home garden	PNMo_6	Moneragala	Home garden
PNMatr_8	Matara	Home garden	PNMo_7	Moneragala	Home garden
PNHam_1	Hambantota	Home garden	PNMo_8	Moneragala	Home garden

PNHam_2	Hambantota	Home garden	PNMo_9	Moneragala	Home garden
PNHam_3	Hambantota	Home garden	PNMo_10	Moneragala	Home garden
PNHam_4	Hambantota	Home garden	PNMo_11	Moneragala	Home garden
PNHam_5	Hambantota	Home garden	PNMo_12	Moneragala	Home garden
PNHam_6	Hambantota	Home garden	PNMo_13	Moneragala	Home garden
PNHam_7	Hambantota	Home garden	PNMo_14	Moneragala	Home garden
PNHam_8	Hambantota	Home garden	PNMo_15	Moneragala	Home garden
PNHam_9	Hambantota	Large scale cultivations	PNMo_16	Moneragala	Home garden
PNHam_10	Hambantota	Large scale cultivations	PNMo_17	Moneragala	Home garden
PNHam_11	Hambantota	Large scale cultivations	PNMo_18	Moneragala	Home garden
PNHam_12	Hambantota	Large scale cultivations	PNMo_19	Moneragala	Home garden
PNHam_13	Hambantota	Large scale cultivations	PNMo_20	Moneragala	Home garden
PNHam_14	Hambantota	Large scale cultivations	PNMo_21	Moneragala	Home garden
PNHam_15	Hambantota	Large scale cultivations	PNMo_22	Moneragala	Home garden
PNHam_16	Hambantota	Large scale cultivations	PNMo_23	Moneragala	Home garden
PNHam_17	Hambantota	Large scale cultivations	PNMo_24	Moneragala	Home garden
PNHam_18	Hambantota	Home garden	PNMo_25	Moneragala	Home garden
PNHam_19	Hambantota	Home garden	PNMo_26	Moneragala	Home garden
PNHam_20	Hambantota	Home garden	PNMo_27	Moneragala	Home garden
PNHam_21	Hambantota	Home garden	PNMo_28	Moneragala	Home garden
PNKeg_1	Kegalle	Home garden	PNMo_29	Moneragala	Home garden
PNKeg_2	Kegalle	Home garden	PNMo_30	Moneragala	Home garden
PNKeg_3	Kegalle	Home garden	PNRa_1	Ratnapura	Home garden
PNKeg_4	Kegalle	Home garden	PNRa_2	Ratnapura	Home garden

PNKeg_5	Kegalle	Home garden	PNRa_3	Ratnapura	Home garden
PNKeg_6	Kegalle	Home garden	PNRa_4	Ratnapura	Home garden
PNKeg_7	Kegalle	Home garden	PNRa_5	Ratnapura	Home garden
PNKeg_8	Kegalle	Home garden	PNRa_6	Ratnapura	Home garden
PNKeg_9	Kegalle	Home garden	PNRa_7	Ratnapura	Home garden
PNKeg_10	Kegalle	Home garden	PNRa_8	Ratnapura	Home garden
PNKeg_11	Kegalle	Home garden	PNRa_9	Ratnapura	Home garden
PNKeg_12	Kegalle	Home garden	PNRa_10	Ratnapura	Home garden
PNKeg_13	Kegalle	Home garden	PNRa_11	Ratnapura	Home garden
PNKeg_14	Kegalle	Home garden	PNRa_12	Ratnapura	Home garden
PNMatl_1	Matale	Home garden	PNRa_13	Ratnapura	Home garden
PNMatl_2	Matale	Home garden	PNRa_14	Ratnapura	Home garden
PNMatl_3	Matale	Home garden	PNRa_15	Ratnapura	Home garden
PNMatl_4	Matale	Home garden	PNRa_16	Ratnapura	Home garden
PNMatl_5	Matale	Home garden	PNRa_17	Ratnapura	Panniyur-1
PNMatl_6	Matale	Home garden	PNRa_18	Ratnapura	Large scale cultivations
PNMatl_7	Matale	Home garden	PNRa_19	Ratnapura	Large scale cultivations
PNMatl_8	Matale	Home garden	PNRa_20	Ratnapura	Large scale cultivations
PNMatl_9	Matale	Large scale cultivations	PNRa_21	Ratnapura	Large scale cultivations
PNMatl_10	Matale	Hybrid "Dingi_Rala"	PNRa_22	Ratnapura	Large scale cultivations
PNMatl_11	Matale	Hybrid "Bootawe_Rala"	PNRa_23	Ratnapura	Large scale cultivations
PNMatl_12	Matale	Hybrid "Kohukumbure_ Rate_Rala"	PNRa_24	Ratnapura	Large scale cultivations
PNMatl_13	Matale	Large scale cultivations	PNRa_25	Ratnapura	Large scale cultivations

PNMatl_14	Matale	Large scale cultivations	PNRa_26	Ratnapura	Large scale cultivations
PNMatl_15	Matale	Large scale cultivations	PNRa_27	Ratnapura	Large scale cultivations
PNMatl_16	Matale	Large scale cultivations	PNRa_28	Ratnapura	Large scale cultivations
PNMatl_17	Matale	Large scale cultivations	PNRa_29	Ratnapura	Large scale cultivations
PNMatl_18	Matale	Large scale cultivations	PNRa_30	Ratnapura	Large scale cultivations
PNMatl_19	Matale	Large scale cultivations	PNRa_31	Ratnapura	Large scale cultivations
PNMatl_20	Matale	Large scale cultivations	PNRa_32	Ratnapura	Large scale cultivations
PNMatl_21	Matale	Large scale cultivations	PNRa_33	Ratnapura	Home garden
PNMatl_22	Matale	Large scale cultivations	PNRa_34	Ratnapura	Home garden
PNMatl_23	Matale	Large scale cultivations	PNRa_35	Ratnapura	Home garden
PNMatl_24	Matale	Large scale cultivations	PNRa_36	Ratnapura	Home garden
PNMatl_25	Matale	Large scale cultivations	PNRa_37	Ratnapura	Home garden
PNMatl_26	Matale	Large scale cultivations	PNRa_38	Ratnapura	Home garden
PNMatl_27	Matale	Large scale cultivations	PNRa_39	Ratnapura	Home garden
PNMatl_28	Matale	Large scale cultivations	PNRa_40	Ratnapura	Home garden
PNMatl_29	Matale	Large scale cultivations	PNRa_41	Ratnapura	Home garden
PNMatl_30	Matale	Large scale cultivations	PNRa_42	Ratnapura	Home garden
PNMatl_31	Matale	Large scale cultivations			
