# Supplementary material for: Genetic diversity and population structure of Piper nigrum (black pepper) accessions based on next-generation SNP markers
Source: PLoS One. 2024 Jun 26;19(6):e0305990. doi: 10.1371/journal.pone.0305990 (PMC11207170; doi:10.1371/journal.pone.0305990)
Supplement: S2 Table — (PDF) [file pone.0305990.s002.pdf]

S2 Table The summary statistics for quality filtered reads of 175 *Piper nigrum* accessions

	**Total reads**	**Read 1**	**Read 2**
Minimum	1,619,259	808,488	810,771
Maximum	20,815,475	10,431,628	66,117,685
Average	11,291,967	5,643,165	5,988,787
