# Supplementary material for: Genetic diversity and population structure of Piper nigrum (black pepper) accessions based on next-generation SNP markers
Source: PLoS One. 2024 Jun 26;19(6):e0305990. doi: 10.1371/journal.pone.0305990 (PMC11207170; doi:10.1371/journal.pone.0305990)
Supplement: S3 Table — (PDF) [file pone.0305990.s003.pdf]

S3 Table Analysis of molecular variance (AMOVA) among structure-based two subpopulations of *Piper nigrum* accessions

**Source of variation**	**Sum of squares**	**Variance components**	**Percentage variation**	F_{ST}	***p*-value**
Among populations	8453.078	5.876	0.07	0.00073	<0.0001
Within populations	2521661.762	8019.654	99.93		
Total	2530114.840	8025.52942			

F_{ST}, fixation index
